# Brazilian vascular surgeons experience during the coronavirus (COVID-19) pandemic

**DOI:** 10.1177/1708538120954961

**Published:** 2020-10-06

**Authors:** Rafael D Malgor, Marcone Lima Sobreira, Nicolas J Mouawad, Adam P Johnson, Max V Wohlauer, Sheila M Coogan, Robert F Cuff, Dawn M Coleman, Malachi G Sheahan, Karen Woo, Sherene Shalhub

**Affiliations:** 1 Anschutz Medical Center, Division of Vascular Surgery and Endovascular Therapy, The University of Colorado, Aurora, CO, USA; 2 Division of Vascular Surgery, Botucatu School of Medicine, Sao Paulo State University, Botucatu, Brazil; 3 Vascular & Endovascular Surgery, McLaren Health System, Bay City, MI, USA; 4 New York/Presbyterian Weill Cornell School of Medicine, New York, NY, USA; 5 Department of Cardiovascular Surgery, University of Texas at Houston, Houston, TX, USA; 6 Vascular Surgery, Spectrum Health Medical Group, Grand Rapids, MI, USA; 7 Department of Surgery, University of Michigan, Ann Arbor, MI, USA; 8 Division of Vascular and Endovascular Surgery, Louisiana State University Health Sciences Center, New Orleans, LA, USA; 9 Division of Vascular Surgery. University of California Los Angeles, Los Angeles, CA, USA; 10 Division of Vascular Surgery, Department of Surgery, University of Washington, Seattle, WA, USA

**Keywords:** COVID-19, vascular surgery practice, brief COPE, GAD-7

## Abstract

**Background:**

The COVID-19 pandemic has made a significant impact on all spheres of society. The objective of this study was to examine the impact of COVID-19 on the practices, finances, and social aspects of Brazilian vascular surgeons’ lives.

**Methods:**

This is a descriptive analysis of the responses from Brazilian vascular surgeons to the cross-sectional anonymous Society for Vascular Surgery Wellness Task Force Pandemic Practice, Anxiety, Coping, and Support Survey for Vascular Surgeons disseminated 14–24 April 2020. Survey dissemination in Brazil occurred mainly via the Brazilian Society of Angiology and Vascular Surgery (SBACV) and social media. The survey evaluated the impact of the COVID-19 pandemic on vascular surgeons’ lives by assessing COVID-19-related stressors, anxiety using theGeneral Anxiety Disorder (GAD)-7 scale, and coping strategies using the Brief Coping Orientation to Problems Experienced (Brief-COPE) inventory.

**Results:**

A total of 452 responses were recorded from Brazil, with 335 (74%) respondents completing the entire survey. The majority of respondents were males (*N* = 301, 67%) and practiced in an urban hospitals. The majority of respondents considered themselves at high risk to be infected with COVID-19 (*N* = 251, 55.8%), and just over half the respondents noted that they had adequate PPE at their primary hospital (*N* = 171, 54%). One hundred and nine (35%) surgeons confirmed that their hospitals followed professional surgical society guidelines for prioritizing surgeries during the pandemic. At the time of the survey, only 33 (10%) surgeons stated they have pre-operative testing of patients for COVID-19 available at their hospital. Academic vascular surgeons reported being redeployed more often to help with other non-vascular duties compared to community-based or solo practitioners (43% vs. 30% vs. 21% respectively, *P* = .01). Severe anxiety due to pandemic-related financial concerns was similar in those surgeons practicing solo compared to those in community- or academic-based/group practice (46% vs. 38% vs. 22%; *P* = .54). The respondents reported their anxiety levels as mild based on the stressors investigated instead of moderate-severe (54% vs. 46%; *P* = .04). Social media was utilized heavily during the pandemic, with video gatherings being the most commonly used tool (76%). Self-distraction (60%) and situational acceptance (81%) were the most frequently reported coping mechanisms used among Brazilian vascular surgeons.

**Conclusion:**

The COVID pandemic has greatly affected healthcare providers around the world. At the time of this survey, Brazilian vascular surgeons are reporting low anxiety levels during this time and are using mostly active coping mechanisms.

## Introduction

The COVID-19 pandemic caused by the severe acute respiratory syndrome coronavirus 2 (SARS-CoV-2) has made a significant impact globally on all spheres of society.^1^,^2^ Flu-related pandemic and outbreaks are not infrequent in modern history as seen with the Influenza H1N1 pandemic in 2009, and the Middle East Respiratory Syndrome coronavirus (MERS-CoV) outbreak in 2015.^3^,^4^ However, the infection and death rates secondary to SARS-CoV-2 have reached alarming numbers.^5^

The response to and impact of the pandemic has differed from country to country. Major world economic powers, such as Italy, Spain, France, the UK, China, and the USA were considerably affected despite their robust public health infrastructure and technological development.^1^,^2^

Brazil has one of the largest practicing vascular surgery communities in the world, with around 3500 board-certified vascular surgeons. Brazil is the fifth-largest country, the ninth-largest economy by nominal gross domestic product, and the eighth-largest nation by purchasing power parity in the world. As a developing country, healthcare is universal in Brazil, with basic access provided to all citizens. However, complementary private healthcare systems coexist with the unified public healthcare system, which accentuate the separation between the rich and the poor.^6^ The objective of this study was to analyze the survey responses from vascular surgeons in Brazil to understand the impact of the COVID-19 pandemic on these individuals during the time period of 10–24 April 2020.

## Methods

This is a subset analysis of the cross-sectional anonymous Society for Vascular Surgery (SVS) Wellness Task Force Pandemic Practice, Anxiety, Coping, and Support Survey for Vascular Surgeons, which was made available between 14 April 2020 and 24 April 2020 inclusive. Institutional review board approval was granted through the University of Washington Human Subjects Division (IRB#09926) as a minimal risk project with consent waiver as the input provided by the respondents is not identifiable. All participants implicated consent by replying to the survey. The survey evaluated the impact of the COVID-19 pandemic on vascular surgeons’ clinical practices, as well as their degree of anxiety, measured by the GAD-7 scale.^7^ Coping strategies were also assessed using the 28-item Brief Coping Orientation to Problems Experienced (Brief-COPE) inventory(Brief-COPE).^8^

The survey (online Appendix 1) was disseminated through the SVS media channels the SVS Connect and the Pulse. Social media, such as Twitter, WhatsApp, and Facebook were also utilized to distribute the survey. Dissemination in Brazil also occurred through the Brazilian Society of Angiology and Vascular Surgery (SBACV). This was accomplished through two email distributions sent to more than 3500 members on 16 April 2020 and 22 April 2020.

### Statistical analysis

Categorical data are reported as numbers and percentages. Continuous data are presented as means and standard deviation of the mean or median and ranges or interquartile range (IQR) where appropriate. Measures of central tendency of numerical data were compared using the parametric Student’s *t*-test for normally distributed data and non-parametric Wilcoxon rank-sum test for non-normal data. Categorical data were compared using the Pearson Chi-square test. Data were analyzed using SPSS 19.0 for Windows (SPSS, Inc., Chicago, IL, USA).

## Results

A total of 452 responses were received from Brazil for an estimated 13% response rate. All but 10 (2%) practicing vascular specialists responding to the survey were vascular surgeons. The majority of respondents were males (*N* = 301, 67%). The three most common types of practices reported were academic, private sector, and government. The vast majority of respondents practiced in an urban hospital with <1% practicing in the rural setting. Years in practice were equally distributed among the respondents. Further information on type of practice, size of hospital, and leadership positions is shown in Table 1.

**Table 1.** Respondents demographics and professional experience.

*N* = 452	*N* (%)
Respondents	
Vascular surgeons	442 (97.8)
Angiology	7 (1.5)
Interventional radiology	3 (.6)
Gender	
Male	301 (66.6)
Female	151 (33.4)
Years in practice (*N* = 452)	
In practice less than 10 years	173 (38.3)
In practice between 10 and 20 years	133 (29.4)
In practice greater than 20 years	146 (32.3)
What is the practice type were you work primarily?	
Academic or government run^a^	96 (21.2)
Community/Group practice^b^	224 (49.6)
Solo or Outpatient practice only	132 (29.2)
Type of hospital (*N* = 314)	
Urban teaching	132 (42)
Urban non-teaching	179 (57)
Rural teaching	2 (0.6)
Rural non-teaching	1 (0.4)
Size of hospital (*N* = 314)	
<50 beds	13 (4)
50–99 beds	34 (10)
100–200 beds	75 (234)
201–300 beds	49 (16)
301–400 beds	43 (14)
>400 beds	75 (24)
Don’t know	25 (8)
Institutional leadership (*N* = 405)	154 (38)
Practice at more than one hospital (*N* = 315)	243 (77)

^a^Government=national, public healthcare system “Sistema Único de Saúde”.

^b^Combined multi-specialty, vascular surgery group, and community-based practices.

### Practice impact and exposure to COVID-19

At the time of the survey, most respondents reported that elective cases were cancelled (92.5%) and that they had available beds in their intensive care units (ICUs, 82.6%). One hundred and nine (35%) surgeons confirmed their hospitals had adopted the American College of Surgeons/Society for Vascular Surgery guidelines for prioritizing surgeries during the pandemic. Just over half of the respondents (*N* = 166, 53%) reported receiving regular updates about the status of COVID-19 at their hospital. Academic vascular surgeons reported being redeployed more often to help with other non-vascular duties compared to community-based or solo practitioners (42.9% vs. 29.6% vs. 20.9%, respectively, *P* = .01, Table 2).

**Table 2.** COVID-19 occupational exposure, practice impact, and hospital support by type of practice.

*N* (%)	All	Academic or government run	Community/group practice	Solo or outpatient practice only	*P*-value
COVID-19 Occupational Exposure					
Number of respondents	452	96	224	132	
Operated on a patient with conﬁrmed COVID-19 infection	26 (5.8)	6 (6.3)	16 (7.1)	4 (3)	0.266
Operated or performed a procedure on patient with conﬁrmed COVID-19 infection	66 (14.6)	18 (18.8)	40 (17.9)	8 (6.1)	0.004
Operated or performed a procedure on patient who was later diagnosed with a COVID-19 infection	51 (11.3)	15 (15.6)	28 (12.5)	8 (6.1)	0.057
Personally considered at “high” risk for COVID-19 infection	251 (55.8)	63 (66.3)	124 (55.6)	64 (48.5)	0.028
ICU availability					
Number of respondents	281	70	141	61	
There are AVAILABLE beds in the ICU	232 (82.6)	55 (78.6)	116 (82.3)	61 (87.1)	0.406
ICUs are full, patients are boarding in the Emergency department	43 (15.3)	12 (17.1)	51 (14.9)	10 (14.3)	0.879
ICUs are full, patients are boarding in the PACU and operating rooms	12 (4.3)	2 (2.9)	7 (5.1)	3 (4.3)	0.776
Surgery Schedule					
Number of respondents	321	75	153	93	
Elective Surgeries cancelled	297 (92.5)	67 (89.3)	142 (92.8)	88 (94.6)	0.424
Ambulatory clinic schedules					
Number of respondents	320	75	152	93	
Regular clinic/ambulatory centers hours	63 (19.7)	13 (17.3)	323(21.7)	17 (18.3)	0.679
Limited clinic/ambulatory centers hours	188 (58.8)	46 (61.3)	85 (55.9)	57 (61.3)	0.62
Patient visits via telehealth	62 (19.4)	18 (24)	29 (19.1)	15 (16.1)	0.435
No clinic and no telehealth	54 (16.4)	12 (16)	27 (17.8)	15 (16.1)	0.922
Call schedule changes					
Number of respondents	312	74	147	91	
Call schedule changes	234 (75)	50 (68.9)	115 (78.2)	68 (74.7)	0.32
Duty changes					
Number of respondents	303	70	142	91	
Assist in duties other than those of a vascular surgeon	91 (30)	30 (42.9)	42 (29.6)	19 (20.9)	0.01
The primary hospital or facility where you work has					
Number of respondents	321	75	153	93	
Pre-operative testing of patients for COVID-19	33 (10.3)	9 (12)	15 (9.8)	9 (9.7)	0.854
COVID-19 Operating Room protocols	235 (73.2)	56 (74.7)	113 (73.9)	66 (71)	0.839
Adheres to ACS/SVS guidelines for allowable surgeries during COVID-19	110 (34.3)	33 (44)	51 (33.3)	26 (28)	0.088
Adequate personal protective equipment (PPE)	172 (53.5)	35 (46.7)	91 (59.5)	46 (49.5)	0.122
Regular updates about the status of COVID-19 at the hospital	168 (52.3)	43 (57.3)	85 (55.6)	40 (43)	0.099
Opportunities to interact with leadership and provide feedback/ask questions	137 (42.7)	37 (49.3)	64 (41.8)	36 (38.7)	0.368
Transparency from leadership about COVID management and planning	145 (45.2)	34 (45.3)	68 (44.4)	43 (46.2)	0.963

A small portion of the vascular surgeons have either operated on or placed a central venous catheter (CVC) in a patient with a confirmed COVID-19 infection (*N* = 66, 14.6%). In terms of perioperative precautions, among those who operated on patients with COVID-19 (*N* = 26), 92% (*N* = 24) used N95 masks, and 11% (*N* = 3) used Powered Air-Purified Respirator during operations. Over half reported that they had adequate PPE at their primary hospital (*N* = 171, 54%). Among those who had performed central venous access (CVC) placement on COVID-19 patients, most noted they had adequate PPE during the procedures (*N* = 51, 84%).

In some circumstances, (*N* = 51, 11.3%) vascular surgeons reported that they operated or performed a procedure on a patient who was later diagnosed with COVID-19 infection. Of those 51, 34 (67%) continued to work, 7 (14%) self-quarantined, and 6 (12%) were tested for COVID-19. Only 33 (10.3%) surgeons stated pre-operative testing of patients for COVID-19 was available at the time of the survey. The majority of respondents considered themselves at high-risk to be infected with COVID-19 (*N* = 251, 55.8%, Table 2). Nine (2%) respondents had tested positive for COVID-19.

### Personal impact

**Figure 1.**
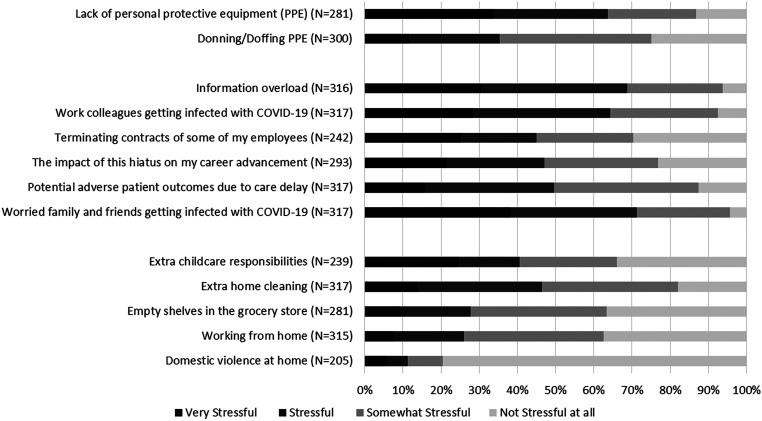
Magnitude of stress reported by vascular surgeons towards occupational and personal COVID-19-related stressors.

The vast majority of respondents (*N* = 395, 88%) reported changes in daily routine and social life. The majority of respondents (352, 78%) stated they used a separate changing area at home after work, but continued to use the shared spaces. Thirty-six (8%) stated they stayed in a separate room at home while 6 (1.3%) remained in a hotel or the hospital. Concerns of becoming disabled or dying during the pandemic prompted 130 (30%) Brazilian vascular surgeons to review or make a living will, 89 (21%) to designate or re-discuss their medical power of attorney, 65 (15%) to review or to apply for disability/life insurance, and 145 (34%) to discuss dying with family or friends. Figure 1 summarizes the magnitude of stress reported by the respondents associated with occupational and personal COVID-19-related stressors.

**Figure 2.**
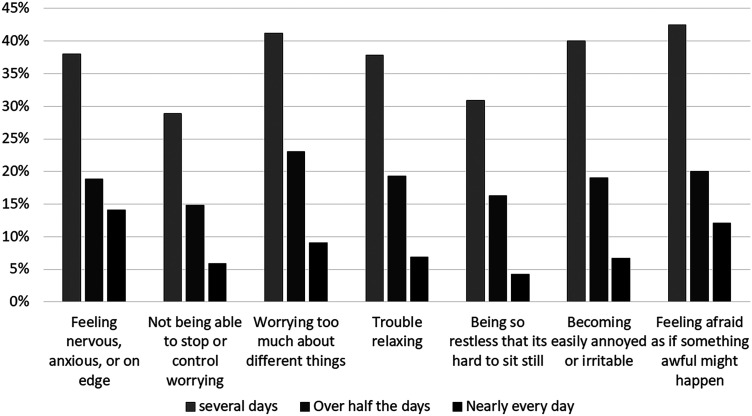
Responses to the generalized anxiety disorder 7-item (GAD-7) scale asking “Over the last 2 weeks, how often have you been bothered by the following problems?” (*N* = 396).

**Figure 3.**
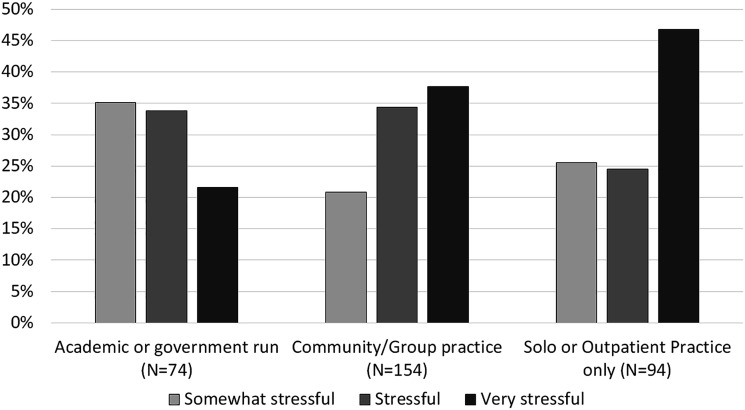
The degree of stress associated with COVID-19-related financial concerns by vascular surgeons (*N* = 322). The difference in self-reported stress level was statistically significant by type of practice (*P* = .024).

Of the 315 (71.3%) respondents who replied to the question regarding COVID-19-related financial concerns, the majority (93.3%) found it stressful (Figure 2). Moreover, 151 (47%) vascular surgeons practicing in solo or exclusively outpatient practices reported that financial concerns were causing them severe stress as opposed to 122 (38%) of surgeons working in community/group practices and 68 (22%) of those working for academic or government institutions (*P* = 0.01). Less than 10% of the respondents in each group reported the financial impact of the pandemic to not be stressful at all. The GAD-7 scale was completed by 405 (90.7%) respondents. Nearly a third reported no anxiety (*N* = 136, 33.6%), a third (*N* = 153, 38%) reported mild anxiety, and the rest had moderate anxiety (*N* = 76, 18.8). Responses to the GAD-7 questionnaire are shown in Figure 3.

**Figure 4.**
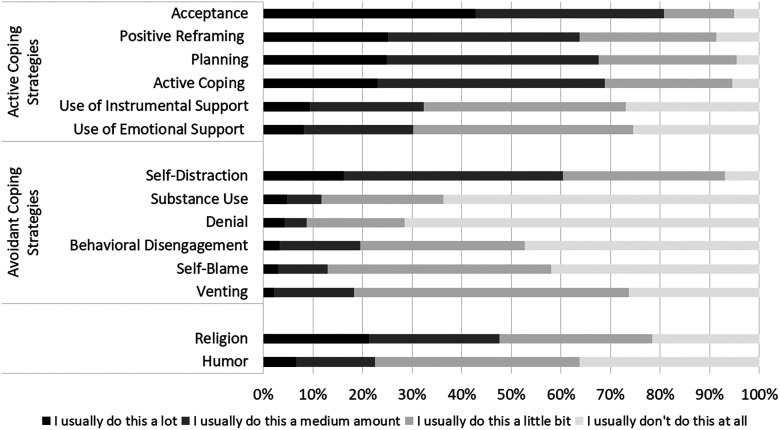
Coping strategies used by the vascular surgeons in Brazil (*N* = 334) as measured by the Brief Coping Orientation to Problems Experienced (Brief-COPE) inventory.

**Figure 5.**
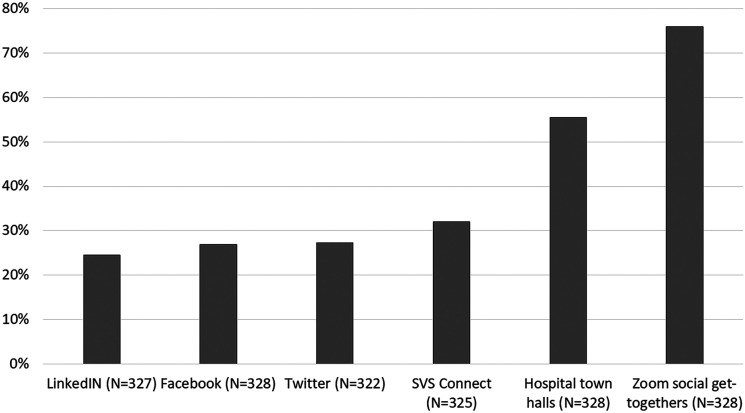
Usefulness of peer to peer support via social media platforms and virtual meetings.

The Brief-COPE inventory was completed by 334 (75.6%) of the respondents. The most commonly reported avoidant coping strategies were self-distraction (60%), followed by venting (73%). The most commonly reported active coping strategies were acceptance, used by 95% of the respondents and planning by 96% (Figure 4**)**. Several social media platforms were utilized by 325 (97%) respondents during the pandemic. These are avenues to maintain interpersonal relationships and potentially cope with stressors. The most commonly used was a video chat for remote gatherings followed by hospital Town Halls (249/328, 76% and 182/328, 55%, respectively, Figure 5).

### Differences in the impact of COVID-19 in Brazil and in the USA

At the timing at this survey, data from several countries were reported in the global survey. Differences between the USA and Brazil were noticeable with significantly more female Brazilian vascular surgeons responding to the survey than female American vascular surgeons (146, 33% vs. 134, 26%; *P* = .016). In addition, there were a significantly higher number of vascular surgeons practicing in teaching hospital and in urban areas in Brazil compared to the USA (134, 43% vs. 130, 26%; *P* < .001 and 311, 99% vs. 437, 89%; *P* < .001).

Operating on patients with confirmed COVID-19 was more commonly reported by Americans (95, 18% vs. 24, 5%; *P* < .001). American vascular surgeons also reported operating on more patients who later test positive for COVID (96, 18% vs. 49, 11%; *P* = 0.003). Although the American vascular surgeon respondents were more exposed to patients with COVID, more Brazilian vascular surgeons responding to the survey considered themselves at high risk to get infected with COVID (243, 55% vs. 147, 28%; *P* < .001). Intensive care unit (ICU) beds were reported to be similarly available in both countries (218, 79% vs. 402, 84%; *P* = 0.082). Whenever ICUs were full, patients were boarded in emergency departments, post-anesthesia care units (PACU) or operating rooms. Patients were found to be predominantly boarded in Emergency departments in Brazil (41, 15% vs. 25, 5%; *P* < .001), whereas more patients were boarded in PACUs and operating rooms in the USA (53, 11% vs. 12, 4%; *P* = 0.002).

Preparedness and response to a possible illness and death varied significantly between Brazil and the USA. Brazilian vascular surgeons reported being more prone to make a living will (130, 32% vs. 78, 15%; *P* < .001), to assign a power of attorney (87, 22% vs. 66, 13%; *P* < .001), or to discuss health insurance coverage compared to their American counterparts (65, 16% vs. 47, 9%; *P* < .001). A third of the respondents in both countries reported discussing death with family and friends. Table 3 summarizes the main comparisons between Brazilian and American vascular surgeon responses.

**Table 3.** Comparison between Brazilian and American vascular surgeons’ responses.

	Brazil	The USA	*P*-value
Gender			
Male	296 (67)	391 (73.1)	0.016
Female	146 (33)	134 (25.6)	
Years in practice			
In practice less than 10 years	169 (38.2)	223 (41.7)	0.508
In practice between 10 and 20 years	132 (29.9)	156 (29.2)	
In practice greater than 20 years	141 (31.9)	156 (29.2)	
Hospital type			
Teaching hospital	134 (42.7)	130 (26.4)	<.001
Urban hospital	311 (99)	437 (88.6)	<.001
Occupational Exposure			
Operated on a patient with conﬁrmed COVID-19 infection	24 (5.4)	95 (17.8)	<.001
Operated or performed a procedure on patient with conﬁrmed COVID-19 infection	63 (14.3)	131 (24.5)	<.001
Operated or performed a procedure on patient who was later diagnosed with a COVID-19 infection	49 (11)	96 (17.9)	0.003
Personally considered at “high” risk for COVID-19 infection	243 (55.2)	147 (27.6)	<.001
Intensive care unit (ICU) availability			
There are AVAILABLE beds in the ICU	218 (78.7)	402 (83.8)	0.082
ICUs are full, patients are boarding in the Emergency department	41 (14.8)	25 (5.2)	<.001
ICUs are full, patients are boarding in the PACU and operating rooms	12 (4.3)	53 (11)	0.002
Surgery Schedule			
Number of respondents	314	493	
Elective Surgeries cancelled	291 (92.7)	452 (91.7)	0.611
Call schedule changes			
Number of respondents	306	492	
Call schedule changes	229 (74.9)	216 (43.9)	<.001
Duty changes			
Number of respondents	303	492	
Assist in duties other than those of a vascular surgeon	91 (30)	171 (34.8)	0.169
The primary hospital or facility where you work has			
Pre-operative testing of patients for COVID-19	33 (10.5)	243 (49.4)	<.001
COVID-19 Operating Room protocols	232 (73.9)	451 (91.7)	<.001
Adheres to ACS/SVS guidelines for allowable surgeries during COVID-19	109 (34.7)	425 (86.4)	<.001
Adequate personal protective equipment (PPE)	171 (54.5)	396 (80.5)	<.001
Hospital updates	166 (52.9)	437 (88.8)	<.001
Opportunities to interact with leadership	137 (43.6)	356 (72.4)	<.001
Transparency from leadership	144 (45.9)	343 (67.9)	<.001
Personal impact			
Number of respondents	401	522	
Made a living will	130 (32.4)	78 (14.9)	<.001
Assigned a power of attorney	87 (21.7)	66 (12.6)	<.001
Discussed health insurance coverage	65 (16.2)	47 (9)	<.001
Discussed death	140 (34.9)	207 (39.7)	0.14
I personally know someone who died during COVID-19	203 (45.9)	96 (17.9)	<.001
I personally know of a medical provider (physicians, nurses, respiratory therapist) from my hospital who died from COVID-19	134 (30.3)	71 (13.3)	<.001
I have a family member or friend who died of COVID-19	23 (5.2)	31 (5.8)	0.688
Completed GAD-7 survey	396 (89.6)	522 (97.6)	<.001
Completed COPE survey	334 (75.6)	503 (94)	<.001

PACU: Post-anesthesia care unit; GAD-7: generalized anxiety disorder-7.

## Discussion

Brazil has now become the new epicenter of COVID-19 infections with reported 391,222 cases of COVID-19 infection as of 26 May 2020.^9^ The SVS Wellness Task Force Committee aimed to investigate the impact of this pandemic on vascular surgeons’ lives globally. Brazilian vascular surgeons are an integral part of the hospital frontline either by performing direct operations to treat patients with vascular complications secondary to the coronavirus syndrome or by providing intensive care support. Therefore, some unique characteristics of this healthcare system are worth noting.

This finding could be related to the timing of the survey dissemination as Brazil was experiencing the early stage of the COVID-19 surge with 25,262 reported cases at the start of the survey dissemination to 52,995 reported cases by the close of the survey. This of course may change as the case numbers have increased in Brazil.^9^ Brazilian vascular surgeons also reported a stronger, more regular focus on religion as a coping mechanism as opposed to vascular surgeons in the USA. This factor makes the Brazilian data regarding how vascular surgeons report changes in life and stress unique.

The unprecedented magnitude of the COVID-19 pandemic directly affects the world supply chain necessary to contain the infection, save lives, and protect healthcare workers. In several locations, there have been shortages of personal protective equipment (PPE) and life support devices, such as ventilators. The intense physical burden for healthcare providers caring for acutely ill patients with SARS-CoV-2 with limited PPE can generate substantial psychological strain.^10^ This may be translated into states of depression, anxiety, and, later on, post-traumatic stress disorder.^11^ Brazilians who are known for their hospitality and emphasis on regular gatherings with family and friends have reported low anxiety levels at the time of the survey dissemination. These findings could also be related to the pre-surge timing of the survey dissemination. As routine life modification, such as social distancing and lockdowns, become more frequent, the response to stress will likely increase. Our global survey showed a higher level of stress in the US areas severely afflicted by the COVID-19 pandemic, which might also happen in largely populated Brazilian state capitals (i.e. Brasilia, Sao Paulo, Rio de Janeiro).

Fear of being infected or knowing someone who died from COVID-19 was reported by almost half of the respondents. This was a likely motivation for the third of respondents who reported reviewing or making a will—another third of the respondents discussed with family and friends their fear of dying from COVID-19. In light of these fears and uncertainties, personal interaction was a strong need among Brazilian vascular surgeons and video chats were the most utilized social media coping strategy over other websites or messaging platforms. This could be related to the preference of Brazilians for audio- and visual-based interactions instead of messaging.

The Brazilian healthcare system has two sources of funding: public and private. Multi-specialty groups or large insurance companies dominate both systems. Healthcare universal coverage is provided to all Brazilian citizens through a unified medicine system, free of charge, called “Sistema Unico de Saude (SUS)”.^6^ Most of the vascular surgeons that have some form of job agreement with SUS also hold private practice jobs. Similar to the Veterans Affairs system in the USA, Brazilian vascular surgeons who work at SUS facilities can claim a pension based on the duration of their services. Dedicated academic vascular surgeons in Brazil who work preferentially or exclusively at University Hospitals funded by SUS are salaried, and there are no options to earn bonuses based on productivity. On the other spectrum of the Brazilian system are vascular surgeons that work for their own solo company or in small groups of private vascular surgeons that run small enterprises. These system-based differences are important, and they were further explored in the survey based on potential economic vulnerability and stress in those practice types. The overall financial impact of the pandemic was reported to be very stressful by less than half of the respondents. This might be related to the employment landscape in Brazil, where most of the practicing vascular surgeons supplement their own private practice with some sort of academic or government-related employment agreement. Among those vascular surgeons who rely almost exclusively on their individual practice or outpatient procedure revenues, there was a significantly higher level of severe stress. Therefore, we believe solo or private practitioners are more vulnerable to the crisis generated by the COVID-19 pandemic.

A comparison between the Brazilian and the American vascular surgeons’ perceptions of COVID showed significant differences in many points of our survey. Some main differences, such as preparedness for illness and death and the higher boarding of patients in Emergency departments instead of PACUs and operating rooms are likely related to social and cultural differences along with these two countries distinct healthcare systems structure. Further, the timing of survey dissemination both in the USA and in Brazil might have heavily contributed for such differences. Brazil was in a pre-surge phase, while the USA was in its surge phase and was considered the epicenter of the pandemic at the time of the survey.

Our study has several limitations, which we attempted to moderate. Recall bias was mitigated as the survey addresses current feelings and situations at the moment. Acquiescence bias was minimized by limiting agree/disagree selections. Selection bias was possible as those most severely affected either may not have had the time to respond to the survey entirely or may have had an increased desire to detail their experience. Further limitations include non-response bias, although several attempts at amplification of the survey were conducted via social media platforms and outreach from the co-authors. The language barrier should be considered a limitation; however, careful analysis of the complete responses provided by more than 400 Brazilian surgeons showed reasonable English literacy.

## Conclusion

The COVID-19 pandemic has greatly affected healthcare providers around the world. At the time of this survey, Brazilian vascular surgeons have reported only mild effects of the pre-surge phase, and the use of adequate coping mechanisms could be demonstrated.

## References

[1] Signorelli C Scognamiglio T Odone A. COVID-19 in Italy: impact of containment measures and prevalence estimates of infection in the general population. Acta Biomed 2020; 91: 175–179. 32275287 10.23750/abm.v91i3-S.9511 PMC7975916

[2] Ben Abdallah I, L Collegiale A Coscas R, et al. Early experience in Paris with the impact of the COVID-19 pandemic on vascular surgery. J Vasc Surg 2020; 72(1): 373. 10.1016/j.jvs.2020.04.467 PMC7179493 32335307

[3] Faridi U. Middle East respiratory syndrome coronavirus (MERS-CoV): impact on Saudi Arabia, 2015. Saudi J Biol Sci 2018; 25: 1402–1405. 30505188 10.1016/j.sjbs.2016.09.020 PMC6252006

[4] Alberti C Orriols R Manzanera R, et al. Flu and other acute respiratory infections in the working population. The impact of influenza A (H1N1) epidemic. Arch Bronconeumol 2010; 46: 634–639. 20970236 10.1016/j.arbres.2010.09.002

[5] Richardson S Hirsch JS Narasimhan M, et al. Presenting characteristics, comorbidities, and outcomes among 5700 patients hospitalized with COVID-19 in the New York City area. JAMA 2020; 323(20): 2052–2059. 10.1001/jama.2020.6775 PMC7177629 32320003

[6] Carvalho RR Fortes PA Garrafa V. Supplemental care from a bioethical perspective. Rev Assoc Med Bras (1992) 2013; 59: 600–606. 24215665 10.1016/j.ramb.2013.06.017

[7] Spitzer RL Kroenke K Williams JB, et al. A brief measure for assessing generalized anxiety disorder: the GAD-7. Arch Intern Med 2006; 166: 1092–1097. 16717171 10.1001/archinte.166.10.1092

[8] Carver CS Scheier MF Weintraub JK. Assessing coping strategies: a theoretically based approach. J Pers Soc Psychol 1989; 56: 267–283. 2926629 10.1037//0022-3514.56.2.267

[9] “Brazil Ministry of Health”. 2020. https://www.devex.com/organizations/ministry-of-health-brazil-52471

[10] Moccia L Janiri D Pepe M, et al. Affective temperament, attachment style, and the psychological impact of the COVID-19 outbreak: an early report on the Italian general population. *Brain Behav Immun* 2020; 87: 75–79. 10.1016/j.bbi.2020.04.048 PMC7169930 32325098

[11] Lai J Ma S Wang Y, et al. Factors associated with mental health outcomes among health care workers exposed to coronavirus disease 2019. JAMA Netw Open 2020; 3: e203976. 32202646 10.1001/jamanetworkopen.2020.3976 PMC7090843

